# Identification of Novel SNPs in Glioblastoma Using Targeted Resequencing

**DOI:** 10.1371/journal.pone.0018158

**Published:** 2011-06-10

**Authors:** Andreas Keller, Christian Harz, Mark Matzas, Benjamin Meder, Hugo A. Katus, Nicole Ludwig, Ulrike Fischer, Eckart Meese

**Affiliations:** 1 Biomarker Discovery Center Heidelberg, Heidelberg, Germany; 2 Department of Human Genetics, Medical School, Saarland University, Homburg, Germany; 3 Department of Internal Medicine, University of Heidelberg, Heidelberg, Germany; Ohio State University Medical Center, United States of America

## Abstract

High-throughput sequencing opens avenues to find genetic variations that may be indicative of an increased risk for certain diseases. Linking these genomic data to other “omics” approaches bears the potential to deepen our understanding of pathogenic processes at the molecular level. To detect novel single nucleotide polymorphisms (SNPs) for glioblastoma multiforme (GBM), we used a combination of specific target selection and next generation sequencing (NGS). We generated a microarray covering the exonic regions of 132 GBM associated genes to enrich target sequences in two GBM tissues and corresponding leukocytes of the patients. Enriched target genes were sequenced with Illumina and the resulting reads were mapped to the human genome. With this approach we identified over 6000 SNPs, including over 1300 SNPs located in the targeted genes. Integrating the genome-wide association study (GWAS) catalog and known disease associated SNPs, we found that several of the detected SNPs were previously associated with smoking behavior, body mass index, breast cancer and high-grade glioma. Particularly, the breast cancer associated allele of rs660118 SNP in the gene SART1 showed a near doubled frequency in glioblastoma patients, as verified in an independent control cohort by Sanger sequencing. In addition, we identified SNPs in 20 of 21 GBM associated antigens providing further evidence that genetic variations are significantly associated with the immunogenicity of antigens.

## Introduction

Glioblastoma (GBM) is the most frequent and most malignant human primary brain tumor that is characterized by a rapid and invasive growth pattern, high level of cellular heterogeneity and poor prognosis [1], [2]. Although current standard care for glioblastoma patients comprises surgical resection followed by adjuvant radiation therapy and chemotherapy with temozolomide, most patients die within 2 years after diagnosis [3]. Genetic hallmarks of glioblastoma are deletion of p16INK4a, mutation of p53 and PTEN, loss of heterozygosity of chromosome 10q [4] , and gene amplifications including EGFR amplified in 37.5% of GBM [5] . Additional amplification are frequently found at 12q13-21 including CDK4, KUB3 or CYP27B1. The gene amplification have been associated with the overall survival of GBM patients [6] . Besides these common features, a manifold of additional aberrations have been identified for GBM with high-throughput whole genome and whole transcriptome analysis tools, including amplicons detected by aCGH data [6]–[8], aberrant gene expression profiles detected by microarray screenings [9]–[11], deregulated microRNAs [12]–[14] or single nucleotide polymorphisms (SNPs) detected by genome wide association studies (GWAS) [15], [16]. Thus, SNPs in the TERT, RTEL1 and CDKN2B regions were strongly associated with high-grade glioma risk. While chromosomal imbalances and aberrant mRNA and miRNA expression profiles can be identified in tumor cells after tumor formation, putative SNPs that might be associated with an increased risk for developing glioblastoma can be identified prior to tumor formation in DNA from normal samples of a patient. These genetic predispositions offer essential screening tools to identify high-risk populations for specific cancers. Furthermore, the knowledge of such predispositions and of the resulting molecular changes in cellular processes will further our understanding of these tumors and will contribute to a personalized medicine.

For decades, Sanger sequencing was the only reliable method available to sequence DNA fragments with high accuracy. However, the throughput was rather low and sequencing of several hundreds of genes was very expensive. For genome wide identification of DNA sequence variations, new sequencing technologies had to be developed. The three next generation sequencing (NGS) systems that are available up to date are based on different principles, including pyrosequencing [17], sequencing-by-synthesis [18] or sequencing-by-ligation [19]. All three methods have in common that DNA is fragmented and bound to adapter sequences that facilitate singularisation and binding of the DNA on solid surfaces. In case of sequencing-by-synthesis and pyrosequencing, second strand DNA is synthesized with incorporated nucleotides being simultaneously detected using either fluorescently-labeled nucleotides in the first or the pyrophosphate-to-ATP conversion and subsequent luciferase reaction in the latter case. For sequencing-by-ligation, fluorescently labeled specific oligonucleotides are hybridized to the immobilized DNA, scanned and subsequently cleaved to enable a new round of hybridization.

Searching for novel SNPs and variants in genes associated with glioblastoma, we combined the HybSelect sequence capture technology of febit [20], a DNA microarray-based capture technology that has been shown to work efficiently with various human cancer-related genes in the past [21], with the deep sequencing technology of Illumina. We selected 132 GBM-associated genes on different chromosomes, spanning a total of 2.2 MB and generated about 60.000 capture probes spanning a region of interest of 504 kb on a customized enrichment chip. We performed targeted enrichment with genomic DNA isolated both from peripheral blood leukocytes and tumor tissue from two different glioblastoma patients. After sequencing, novel SNPs and variants have been called and the most interesting SNPs have been verified in independent samples by Sanger sequencing.

Finally, we analyzed the identified SNPs for an association with 21 autoantigens, suggested triggering a humoral immune response in glioblastoma patients.

## Results

### Targeted re-sequencing

**Figure 1:**
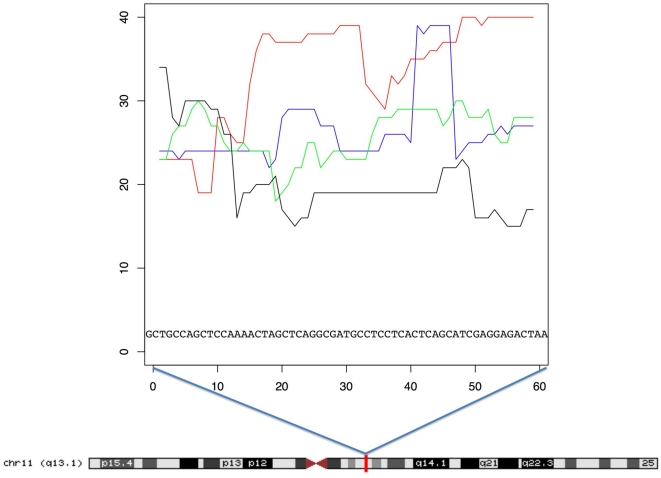
Coverage plot for one exonic part of SART1 for the four control samples. X-axis shows the genomic coordinate inside the exon, y-axis denotes the number of reads covering this position and the four lines correspond to the four genomic samples.

To enrich the genomic DNA for the 132 glioma-associated genes, we applied a microarray based approach as detailed in the Methods section and previously described [21]. The four enriched and sequenced samples are denoted as patient A-leuko, patient B-leuko, patient A-tumor and patient B-tumor. After enrichment of gene sequences with HybSelect, all samples were sequenced on a single lane on an Illumina Genome Analyzer II, generating on average 30.6*10^6^ 50 bp paired-end reads per lane. After excluding artifacts as described in Material and Methods, an average of 16.7*10^6^ paired-end reads remained. After mapping the data to the human genome and excluding reads that map to multiple locations, we obtained on average 3.1*10^6^ reads allowing a 308 fold coverage and a target coverage of approximately 87%. We obtained an up to 844 fold enrichment as compared to a random sequencing. An example of a plot with an exonic part of the SART1 gene and a coverage for the four samples is shown in Figure 1.

### SNP and Indel Analysis

**Figure 2:**
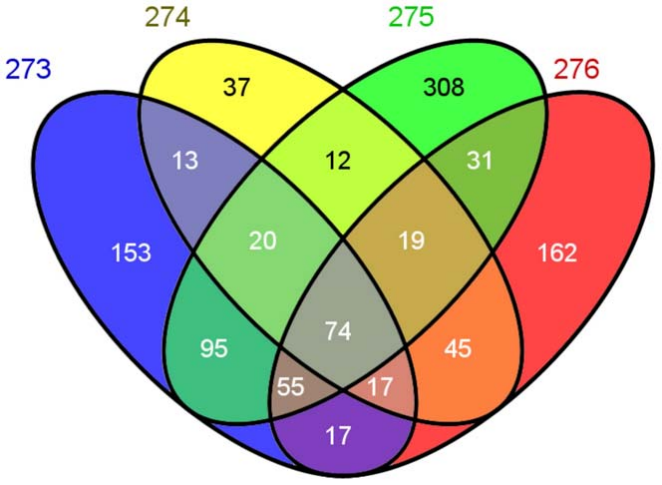
Venn diagram of detected SNPs in the 132 targeted genes in the four samples.

For an in-depth SNP and Indel analysis, the data was remapped using NextGENe (Softgenetics, Pennsylvania, USA). Paired-end reads were mapped against the human genome NCI build 36.1 using NextGENe. The method that is based on hybridization of DNA sequences to complementary oligonucleotides, allows the enrichment of DNA sequences of the target genes, but does not necessarily exclude all sequences similar to the target genes. Therefore, we detected also sequences of genes other than the targeted ones. In total we detected 2753 SNPs or Indels in leukocyte DNA of patient A, 2346 in tumor DNA of patient A, 1527 in leukocyte DNA of patient B, and 2356 in tumor DNA of patient B (Table 1). Considering the 132 GBM associated target genes, we detected 581 SNPs or Indels in leukocyte DNA of patient A, 415 in tumor DNA of patient A, 272 in leukocyte DNA of patient B, and 516 in tumor DNA of patient B. In total, we detected 1317 different SNPs in all four samples with 57 SNPs found in each of the samples, as summarized in the Venn diagram of Figure 2A . Depending on the sample, between 15 and 26% of all detected SNPs and between 34 and 64% of the SNPs in the target genes have already been annotated in the dbSNP. Summarizing all detected SNPs in all four samples, we detected 6196 different SNPs (1057 annotated in dbSNP), including 1317 that mapped to the 132 target genes and 4879 that mapped to the remaining 1030 genes. Table S1 provides the complete list of all detected SNPs in all four samples.

**Table 1:** Overall number of detected SNPs and SNPs detected in target genes.

Sample ID	DNA type	No. of Overall SNPs [thereof known]	No. of SNPs in target genes [thereof known]
patient A	leuko	2753 [444]	581 [197]
patient B	leuko	1527 [235]	272 [112]
patient A	tumor	2346 [613]	415 [265]
patient B	tumor	2356 [420]	516 [239]

Number of known SNPs in brackets.

Performing a pairwise overlap of the four data sets we found more overlaps between the samples of each patient e.g. tumor versus leukocytes, than between the patients. In detail, we found 19.4% and 25.9% overlap for the comparison of tumor versus leukocytes of patient A and patient B, respectively. The overlaps between the blood samples of the two patients were only 18.5%, and between the tumor samples of the two patients 17.3% (Table 2).

**Table 2:** Distribution of detected SNPs among the samples.

sample 1	sample 2	only in sample 1 [n]	only in sample 2 [n]	in both samples [n]	Overlap [%]
patient A leuko	patient A tumor	1924	1517	829	19.4
patient B leuko	patient B tumor	729	1558	798	25.9
patient A leuko	patient B leuko	2086	860	667	18.5
patient A tumor	patient B tumor	1654	1664	692	17.3
patient A leuko	patient B tumor	2044	1647	709	16.1
patient B leuko	patient A tumor	920	1739	607	18.6

To identify SNPs that might indicate tumor specific genetic changes we searched for SNPs that show a different genotype in the leukocytes as compared to the tumor DNA. We found several SNPs with a coverage above 50 including SNP rs715930 in the gene B4GALNT1. For this SNP that is annotated as ‘C’ in the wild type, we found a heterozygous ‘AC’ status in leukocytes of patient A, and an ‘A’ allele in the tumor of this patient. This might either indicate a homozygous ‘AA’ status or potentially a hemizygous status due to loss of heterozygocity in the tumor. SNP rs12172263 in gene sequence LOC643266 is annotated as ‘G’ the reference genome. Likewise, we found a heterozygous ‘TG’ status in the leukocytes of patient A and a ‘T’ allele in the tumor of this patient. Further examples for a heterozygous status in the leukocytes as compared to a hemizygous or homozygous in the according tumor were found for the SNP rs1805414 mapping in PARP1 gene, SNP rs935037 in PAXIP1 gene and SNP rs962976 in MDM1 gene (Table 3).

**Table 3:** SNPs with different genotype in tumor and leukocytes of the same patient.

patient	gene	position	chromosome	reference	sample	coverage	A	C	G	T	genotype	dbSNP	Hgvs*
A	B4GALNT1	56310248	12	C	Leu	154	30;70	0;54	0;0	0;0	AC	rs715930	c.[666C>A]+[ = ]
					Tu	123	61;48	7;6	0;0	0;0	AA	rs715930	c.[666C>A]+[666C>A]
	LOC643266	47216571	22	G	Leu	96	27;0	0;0	69;0	0;0	GA	rs12172263	c.[820+466G>A]+[ = ]
					Tu	115	115;0	0;0	0;0	0;0	AA	rs12172263	c.[820+466G>A]+[820+466G>A]
	LOC644033	4412	8	G	Leu	127	0;0	0;0	21;24	42;40	TG	na	c.[26–95G>T]+[ = ]
					Tu	74	0;0	0;0	7;4	31;32	TT	na	c.[26–95G>T]+[26–95G>T]
	PARP1	224639987	1	A	Leu	102	23;24	0;0	0;55	0;0	GA	rs1805414	c.[852A>G]+[ = ]
					Tu	61	4;4	0;0	11;42	0;0	GG	rs1805414	c.[852A>G]+[852A>G]
B	CTDSP2	56503706	12	C	Leu	118	0;0	51;16	0;0	11;40	CT	na	c.[762C>T]+[ = ]
					Tu	3580	3;0	64;82	4;1	1235;2190	TT	na	c.[762C>T]+[762C>T]
	PAXIP1	154391302	7	A	Leu	114	13;16	0;0	26;59	0;0	GA	rs935037	c.[1440A>G]+[ = ]
					Tu	286	0;0	0;0	64;222	0;0	GG	rs935037	c.[1440A>G]+[1440A>G]
	MDM1	67006894	12	G	Leu	52	27;10	0;0	8;7	0;0	AG	rs962976	c.[308G>A]+[ = ]
					Tu	99	74;8	0;0	13;4	0;0	AA	rs962976	c.[308G>A]+[308G>A]

*HGVC = Human Genome Variation Society.

Supporting Information Legends.

To further elucidate a disease association of the identified SNPs, we computed the overlap between the detected SNPs and the 2967 SNPs reported in the genome-wide association study (GWAS) catalog. We found two SNPs, namely rs6265 that was associated with smoking behavior [22] and body mass index [23] and rs6010620 that was related to high-grade glioma [15].

SNP rs660118 that was found homozygous in both blood and tumor of the two patients, was recently associated with breast cancer [24]. Together with three other SNPs of the SART1 gene (here, aligned NGS reads are shown in Figure 2B), SNP rs660118 constitutes a haplotype that was associated with increased risk of breast cancer. For this haplotype, rs660118 and rs679581 represent the minor alleles (1), and rs754532 and rs735942 the major alleles (0). We found this “1100” haplotype in both blood and tumor of the two patients.

### Sanger verification and independent replication

**Figure 3:**
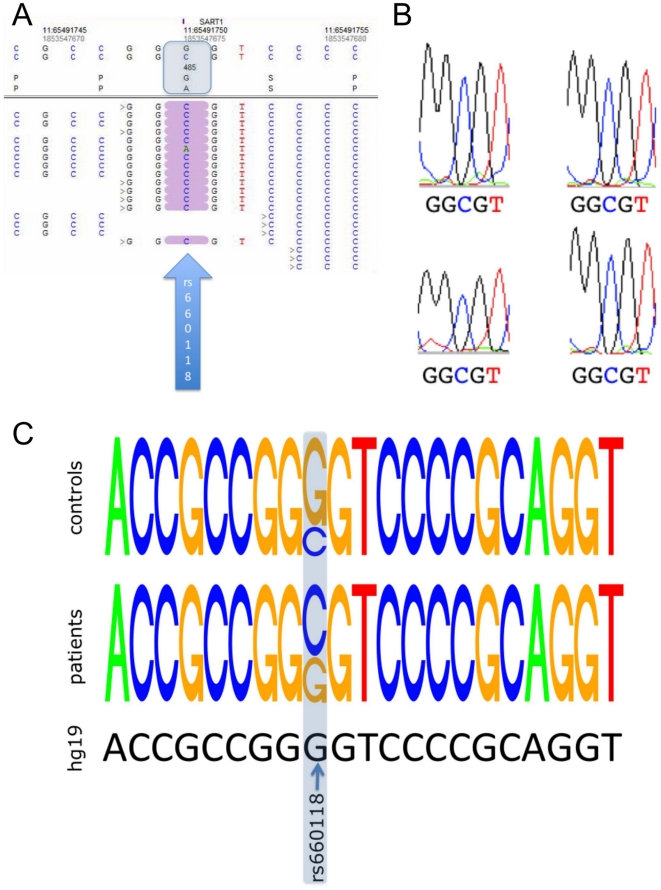
Identification, verification and distribution of the SART1 SNP rs660118. *A*. Next generation sequencing reads of one sample for the SNP rs660118. The blue box at top shows the base and amino acid change. *B*. Sanger traces of the four genomic samples showing a homozygous allele of the SNP in the middle position. *C*. Sequence logo for the part surrounding the SNP computed from Sanger sequencing of the initial samples and the independent cohort. At the bottom, the human reference sequence is presented. Glioblastoma patients show higher counts for the allele ‘C’ as compared to unaffected controls.

For verification purposes we carried out Sanger sequencing of the exons containing the four SNPs. We confirmed the “1100” haplotype in all four samples. This haplotype has not been detected with increased frequency in ten blood samples of glioblastoma patients as compared to ten blood samples of healthy controls. However, including the two samples originally used for SNP identification we found an equal distribution of the SNP rs660118 between glioblastoma patients with homozygous minor allele (C) status, heterozygous (G/C) status, and homozygous major allele (G) status, whereas in the healthy controls we found the wildtype allele in 70% of cases, the rare allele in 10% and the heterozygous status in 20% of cases. Thus the SNP rs660118 that is essential for the “1100” haplotype was correlated with glioblastoma. Sanger Traces are shown in Figure 3A and the results are summarized in the sequence logo in Figure 3B.

### Integrating tumor-associated antigens

Beside the tumor association, we analyzed the identified SNPs for an association with autoantigens. An association between SNPs and immunogenicity of autoantigens was previously suggested [25]. Recently, we reported 21 genes associated with humoral immune response in glioblastoma patients [26]. We detected single nucleotide polymorphisms in at least one of the analyzed samples for 95.2% of these genes (Table S2).

We found five SNPs that were homozygous for alleles that were different from the reference sequence. These homozygous alleles were found in the two patients both in blood and tumor. Specifically, rs4715630 and rs4715631 map within the DST gene, rs176924 in the KIF5B gene, rs2301263 in the GPI gene, and rs41427849 in the ZNF594 gene. We also found five SNPs that were homozygous for the minor alleles in three of the four samples. Specifically, rs1800606 maps within the APBB1 gene, rs4283892 and rs4715626 in DST gene, rs10418774 in the ZNF324 gene and rs4764506 in ING4 gene. In four out of these five SNPs, the leukocytes of at least one patient did not show alleles different from the reference sequence (Table S2). These results provide further evidence that SNPs are associated with the immunogenic antigens.

## Discussion

For four glioblastoma samples we performed targeted resequencing that is increasingly recognized as valuable screening tool for an in-depth analysis of genomic regions while avoiding problems attached to storage and handling of a large collection data that result from whole genome sequencing. With our approach, we could not completely avoid detecting sequences of genes other than the target genes. This might partially be due to sequence similarities between target and non-target genes, e.g. in gene families. An additional limitation is the small number of samples sequenced. However, even this limited number of samples indicates that additional important SNPs are likely to be found in a larger group of patients.

We detected several hundred SNPs mapping to 132 genes in the glioblastoma samples. For several SNPs we found a heterozygous situation in the leukocyte DNA and a homozygous or hemizygous situation in the tumor DNA of the same patient. Unfortunately cytogenetic information on these specific tumor tissues was not available. In general, glioblastoma are genetically highly heterogeneous with multiple chromosomal aberrations including loss of heterozygosity. Losses of chromosomal material occur frequently for chromosomes 6, 9p, 11p, 19q, 22q [27]. Losses of chromosome 10 are associated with primary glioblastoma that occur without any low-grade precursor lesion [2]. Probably, the differences that we found for several SNPs between leukocyte and tumor DNA are due to loss of heterozygosity in the glioblastoma samples.

For several of the identified SNPs there is evidence for an association with cancer. The SNP rs660118 maps within the SART1 (squamous cell carcinoma antigen recognized by T cells) gene that is also known as tumor-rejection antigen in brain tumors [28] and in a range of other tumors including hepatocellular carcinoma [29], colorectal cancer [30], renal cell carcinoma [31], and osteosarcoma [32]. Amplification and overexpression of SART1 occurs in head and neck squamous cell carcinoma (HNSCC) [33]. Functionally, SART1 is required for maintenance of normal mitotic processes with either overexpression or depletion causing mitotic defects including cell cycle arrest and apoptosis [24], [34]. SNPs rs660118 and SNP rs679581 the latter of which also map within the SART1 gene are associated with increased breast cancer risk as shown by the analysis of 798 breast cancer patients and 843 controls [24]. In our study we identified and verified a minor allele of SNP rs660118 in a cohort of glioblastoma and control samples.

Based on the reported evidence that SART1 is a tumor antigen we reviewed our own data on antigens that are reactive with tumor sera [26]. Summarizing the data of more than 400 cases including cancer sera, sera of patients with non-cancer diseases and healthy controls, we found an overall 10% increase of autoantibody response to SART1 in cancer sera as compared to normal controls and non-cancer diseases. It is conceivable that the SNP rs660118 that maps within the exonic sequence of SART1 plays a role in the immunogenicity of this gene. The hypothesis that SNPs are associated with immunogenic antigens was first proposed by Stadler et al. [25]. This idea is also supported by our finding of SNPs in genes previously associated with humoral immune response in glioma patients [26], [35].

In summary, our results provide evidence that targeted resequencing is well suited to detect single nucleotide polymorphisms that may correlate with disease risks. The approach may be especially applicable in personalized medicine to assess the relevant genotype of a patient at reasonable cost. Moreover, combined with multiplexing and customized SNP array design, targeted resequencing also offers the option to validate SNPs detected by GWAS studies in independent sample cohorts.

## Materials and Methods

### Study design

The major aim of our study was the identification of novel SNPs in genes already associated with glioblastoma using a combination of specific target selection and next generation sequencing. We selected a total of 132 genes including 21 genes associated with an anti-tumor humoral immune response in glioma [26] , 55 genes associated with an immune response in glioma or other brain tumors [35]–[37] , and 53 genes shown to be amplified or abnormally expressed in glioblastoma. (For specific details on the selection criteria of specific genes, see Table S1). In addtion we included three genes that were randomly selected for control purposes. Oligonucleotides complementary to the exonic regions of the 132 genes were synthesized on a biochip array as detailed below. This array was used to enrich the target sequences of four genomic DNA samples including tumor and leukocytes of two glioblastoma patients. In detail, genomic DNA was fragmented and adaptors were ligated. The adaptor-DNA-fragments were hybridized to a first array. After stringently washing, bound DNA, which is enriched for the target gene DNA, was eluted as details below. After amplification of the eluted DNA using the adaptor sequence, a second round of enrichment was performed using a second array and the eluted DNA was sequenced. The resulting reads were mapped to the human genome and SNPs were called as detailed below. For analysis we focused on SNPs that were called in multiple samples, that showed differences between leukocytes and tumor of the same patient, and that were called in genes associated with a humoral immune response in brain tumors.

### Microarray Design and Synthesis

Light-activated *in situ* oligonucleotide synthesis on Geniom Biochips was performed as described previously [21]. One Biochip holds eight individual, microfluidic channels each containing an array of 15,624 individual DNA probe features.

For the glioblastoma chip, gene sequences were downloaded from NCBI built 36.1 and 56,184 50mer probes were tiled across the target genes with an average density of 11 bp targeting sense and antisense strand in an alternating manner. For biochip synthesis, they were distributed over 4 arrays. Each exon was covered by at least 20 probes, i.e. small exons were tiled more dense. The full region of interest was 2.2 Mb corresponding to a core target region containing high complexity sequences of 504 Kb.

### DNA sample preparation

5 µg genomic DNA sample were dissolved in 80 µl of water and fragmented for 30 min by sonication at medium intensity (Bioruptor, Diagenode, Liége, Belgium). 5 µl were controlled on a 2% agarose gel for the correct fragment size distribution. Preparation of the paired end adaptor-ligated gDNA library ready for sequencing on an Illumina/Solexa Genome Analyzer II (Illumina, San Diego, CA, USA) was performed according to the manufactures standard protocol. The size fraction of 300–500 bp for gDNA was excised from an agarose gel after the adaptor ligation step and the sample was analyzed by Bioanalyzer 2100 analysis (Agilent), quantified by Nanodrop 1000 UV measurement (Thermo Scientific, Waltham, MA, USA) and stored in EB buffer at −20°C until use.

### Sequence capture protocol

For four arrays, 6 µg adaptor-ligated gDNA library were dried in a speed-vac together with 64 µL febit hybridization booster mix. The dried pellet was dissolved in 100 µl febit Hybmix-4, heated to 95°C for 5 min and placed on ice. The sample mixture was split into four equal parts, placed into the sample loading station of the Geniom RT Analyzer and automatically injected into the microfluidic channels of the biochip. Sample was denatured within the biochip at 80°C for 10 min and hybridized for 16 h at 42°C with active movement of the sample. After hybridization, each array was automatically washed with 6× SSPE at room temperature and 0.5× SSPE at 45°C. Each array was subsequently washed with SSPE-based febit stringent wash buffers 1 and 2 at room temperature. All protocol steps were carried out in a completely automated fashion by the Geniom RT Analyzer instrument without manual interference.

For elution of the enriched samples, arrays were filled with 15 µL of 90% formamide in water each and incubated at 70°C for 30 min. Solution was manually transferred into an reaction tube and dried by vacuum centrifugation in a Speed-Vac at 65°C. After an amplification step according to the Illumina library preparation procedure using primers PE Adapter 1.1 and PE Adapter 2.1 for 30 cycles, the sample was treated like the original library and subjected to a second round of enrichment under the same conditions as before, using a newly synthesized biochip.

Eluted samples were subjected to 10 cycles of PCR according to the conditions described in the Illumina library preparation kit using paired end primers and purified by a Qiagen MinElute PCR purification (Qiagen, Hilden, Germany). Quantification of samples was done by the Quant-It Picogreen assay (Invitrogen, Carlsbad, CA, USA) using the Nanodrop 3300 instrument (Thermo Scientific, Wilmington, DE, USA).

### Paired-End Sequencing

Paired-end sequencing on an Illumina Genome Analyzer II was carried out as described in the manufacturer's manual by Prognosys Inc (La Jolla, CA, USA).

### Data analysis & SNP Verification

Paired-end reads (50 bp in length) were first filtered by removing reads with ambiguous nucleotide calls (3 or more N). Reads from File 1 and File 2 of the two paired end sequences were aligned with target genes by using razerS, which is part of SeqAn, an open source C++ library of efficient algorithms and data structures for the analysis of biological sequences. The parameters used were “-gn 1 -f -r -i 94 -rr 100 -m 10” which allows up to 3 mismatches. The output alignment files were matched for each pair of reads: the two reads were mapped to opposite strands and in correct orientation and the length between the two reads was within 100–500 bp. The fold coverage for each base within the probe regions was calculated for unique reads.

Initial data analysis was carried out with Eland. Re-mapping of the raw reads files was additionally done with NextGENe. The default parameters were used for format conversion and the subsequent alignment, allowing up to two mismatches. Called SNPs with coverage of higher 50 have are considered as SNPs with high confidence, SNPs with coverage of smaller 10 reads have been removed from the consideration.

Subsequent data analysis, including coverage plots, has been carried out using the freely available R [38] and perl software.

Four SNPs were exemplarily verified with Sanger sequencing. We amplified exon 12 of the SART1 gene containing the four SNPs described by Olson et al. [25] and carried out Sanger sequencing using the following primers for amplification and sequencing: forward1-5′-ACTGCGGGGACGGGGT-3′, reverse1-5′-CTTCTCGCCACTGTCTCGCA-3′ (rs660118), respectively: forward2-5′-TCTCTGCAGGAACACCATCA-3′, reverse 2-5′-CGTCCCATCCTTCACACCT-3′ (rs679581, rs754532 and rs735942). Traces have been evaluated with the freely available tool “Mac Sequence View” and alignments have been computed using clustalW.

## Supporting Information

Table S1 **Complete list of the glioblastoma associated genes incuding selection criteria and summary of the detected SNPs in all four samples.**
(XLS)

Table S2 **Detected SNPs, homozygous for minor allele in genes triggering humoral autoimmune response in GBM patients.**
(DOC)
