# Supplementary material for: Identification of Novel SNPs in Glioblastoma Using Targeted Resequencing
Source: PLoS One. 2011 Jun 10;6(6):e18158. doi: 10.1371/journal.pone.0018158 (PMC3112142; doi:10.1371/journal.pone.0018158)
Supplement: Table S2 — Detected SNPs, homozygous for minor allele in genes triggering humoral autoimmune response in GBM patients. (DOC) [file pone.0018158.s002.doc]

Supplementary Table S2: Detected SNPs, homozygous for minor allele in genes triggering humoral autoimmune response in GBM patients

| **Gene** | **SNPS [n]** | Position | **Reference** | Sample* | Coverage [n] | **genotype** | **dbSNP** | HGVC** | **genetic location** |
| --- | --- | --- | --- | --- | --- | --- | --- | --- | --- |
| **DST** | 59 | 56449109 | G | A_Leu | 22 | delGA |  | c.14784-15_14784-14delGA; | Intron |
|  |  |  |  | A_Tu | 11 | delGA |  | c.14784-15_14784-14delGA; |  |
|  |  | 56455042 | A | A_Leu | 25 | delA |  | c.14511-107delA; | Intron |
|  |  | 56473773 | A | A_Leu | 50 | GG | rs4283892 | c.[13005+64A>G]+[13005+64A>G] | Intron |
|  |  |  |  | A_Tu | 26 | GG | rs4283892 | c.[13005+64A>G]+[13005+64A>G] |  |
|  |  |  |  | B_Tu | 33 | GG | rs4283892 | c.[13005+64A>G]+[13005+64A>G] |  |
|  |  | 56482713 | C | A_Tu | 12 | AA | rs9296849 | c.[11889-83C>A]+[11889-83C>A] | Intron |
|  |  | 56489871 | G | A_Leu | 11 | AA | rs4715626 | c.[11466+84G>A]+[11466+84G>A] | Intron |
|  |  |  |  | B_Leu | 11 | AA | rs4715626 | c.[11466+84G>A]+[11466+84G>A] |  |
|  |  |  |  | A_Tu | 28 | AA | rs4715626 | c.[11466+84G>A]+[11466+84G>A] |  |
|  |  | 56525241 | C | A_Leu | 225 | TT | rs4715630 | c.[9417C>T]+[9417C>T] | non-synonymous |
|  |  |  |  | B_Leu | 68 | TT | rs4715630 | c.[9417C>T]+[9417C>T] | M5227I |
|  |  |  |  | A_Tu | 298 | TT | rs4715630 | c.[9417C>T]+[9417C>T] | Spectrin repeat |
|  |  |  |  | B_Tu | 295 | TT | rs4715630 | c.[9417C>T]+[9417C>T] |  |
|  |  | 56525504 | T | A_Leu | 63 | CC | rs4715631 | c.[9154T>C]+[9154T>C] | non-synonymous |
|  |  |  |  | B_Leu | 38 | CC | rs4715631 | c.[9154T>C]+[9154T>C] | A5140T |
|  |  |  |  | A_Tu | 88 | CC | rs4715631 | c.[9154T>C]+[9154T>C] | Spectrin repeat |
|  |  |  |  | B_Tu | 110 | CC | rs4715631 | c.[9154T>C]+[9154T>C] |  |
|  |  | 56533243 | A | B_Tu | 18 | GG | rs2067543 | c.[7419-63A>G]+[7419-63A>G] | Intron |
|  |  | 56545424 | T | B_Tu | 15 | CC | rs1014309 | c.[6663+80T>C]+[6663+80T>C] | Intron |
|  |  | 56570799 | G | B_Leu | 51 | AA | rs9370541 | c.[5037-36G>A]+[5037-36G>A] | Intron |
|  |  |  |  | B_Tu | 12 | AA | rs9370541 | c.[5037-36G>A]+[5037-36G>A] |  |
|  |  | 56571369 | T | B_Tu | 53 | CC | rs4712138 | c.[4901T>C]+[4901T>C] | non-synonymous Q3722R Spectrin repeat |
|  |  | 56615094 | T | B_Tu | 11 | CC | rs1024195 | c.[1239+73T>C]+[1239+73T>C] | Intron |
| **GFAP** | 33 | 40340213 | G | A_Tu | 30 | CC | rs11558961 | c.[1299+28G>C]+[1299+28G>C] | 3’UTR |
|  |  | 40340368 | G | A_Leu | 17 | AA | rs11651396 | c.[1257-86G>A]+[1257-86G>A] | Intron |
|  |  |  |  | A_Tu | 18 | AA | rs11651396 | c.[1257-86G>A]+[1257-86G>A] |  |
|  |  | 40343618 | G | A_Tu | 26 | CC | rs2289679 | c.[1127-66G>C]+[1127-66G>C] | Intron |
| **TTC3** | 32 | 37382358 | T | A_Tu | 32 | AA | rs2835585 | c.[188-8T>A]+[188-8T>A] | Intron |
|  |  | 37384484 | A | A_Leu | 16 | GG | rs3787780 | c.[481+28A>G]+[481+28A>G] | Intron |
|  |  | 37384586 | A | A_Leu | 13 | GG | rs2835588 | c.[481+130A>G]+[481+130A>G] | Intron |
|  |  | 37389486 | C | A_Leu | 15 | AA | rs2006941 | c.[688-34C>A]+[688-34C>A] | Intron |
|  |  | 37390828 | A | A_Leu | 12 | GG | rs2835600 | c.[846+15A>G]+[846+15A>G] | Intron |
|  |  |  |  | A_Tu | 24 | GG | rs2835600 | c.[846+15A>G]+[846+15A>G] |  |
|  |  | 37390887 | T | A_Leu | 27 | GG | rs2835601 | c.[846+74T>G]+[846+74T>G] | Intron |
| **ADD2** | 19 | 70755515 | G | B_Leu | 22 | insA |  | c.1593-51_1593-50insA; | Intron |
|  |  | 70771418 | A | B_Leu | 24 | delAA |  | c.849+8_849+9delAA; | Intron |
| **KIF5B** | 18 | 32351975 | A | A_Leu | 79 | GG | rs176924 | c.[1725-5A>G]+[1725-5A>G] | Intron |
|  |  |  |  | B_Leu | 28 | GG | rs176924 | c.[1725-5A>G]+[1725-5A>G] |  |
|  |  |  |  | A_Tu | 93 | GG | rs176924 | c.[1725-5A>G]+[1725-5A>G] |  |
|  |  |  |  | B_Tu | 16 | GG | rs176924 | c.[1725-5A>G]+[1725-5A>G] |  |
| **DLD** | 12 | 107319070 | G | A_Tu | 121 | insC;GC |  | c.40+99_40+100insC;c.[40+100G>C]+[=] | Intron |
|  |  |  |  | B_Tu | 27 | insC;GC |  | c.40+99_40+100insC;c.[40+100G>C]+[=] |  |
|  |  | 107329898 | T | A_Leu | 14 | CC | rs10250718 | c.[199-108T>C]+[199-108T>C] | Intron |
|  |  |  |  | A_Tu | 14 | CC | rs10250718 | c.[199-108T>C]+[199-108T>C] |  |
|  |  | 107331236 | T | A_Leu | 20 | delT |  | c.338+8delT; | Intron |
|  |  | 107333035 | T | A_Leu | 11 | CC | rs10263341 | c.[439-7T>C]+[439-7T>C] | Intron |
|  |  |  |  | A_Tu | 35 | CC | rs10263341 | c.[439-7T>C]+[439-7T>C] |  |
|  |  | 107343182 | T | A_Tu | 63 | delT |  | c.685-5delT; | Intron |
|  |  | 107347427 | C | A_Tu | 18 | TT | rs4518 | c.[1531+487C>T]+[1531+487C>T] | 3’UTR |
|  |  |  |  | B_Tu | 32 | TT | rs4518 | c.[1531+487C>T]+[1531+487C>T] |  |
| **GPI** | 11 | 39576846 | A | A_Tu | 14 | GG | rs8108038 | c.[1063+35A>G]+[1063+35A>G] | Intron |
|  |  |  |  | B_Tu | 36 | GG | rs8108038 | c.[1063+35A>G]+[1063+35A>G] |  |
|  |  | 39582162 | G | A_Leu | 17 | AA | rs2301263 | c.[1399+82G>A]+[1399+82G>A] | Intron |
|  |  |  |  | B_Leu | 34 | AA | rs2301263 | c.[1399+82G>A]+[1399+82G>A] |  |
|  |  |  |  | A_Tu | 69 | AA | rs2301263 | c.[1399+82G>A]+[1399+82G>A] |  |
|  |  |  |  | B_Tu | 153 | AA | rs2301263 | c.[1399+82G>A]+[1399+82G>A] |  |
| **APBB1** | 7 | 6373535 | C | A_Leu | 16 | TT | rs1800606 | c.[1965-28C>T]+[1965-28C>T] | Intron |
|  |  |  |  | A_Tu | 34 | TT | rs1800606 | c.[1965-28C>T]+[1965-28C >T] |  |
|  |  |  |  | B_Tu | 18 | TT | rs1800606 | c.[1965-28C>T]+[1965-28C>T] |  |
| **VIM** | 5 | 17319598 | C | A_Leu | 11 | AA | rs3179873 | c.[1402+322C>A]+[1402+322C>A] | 3’UTR |
| **ZNF594** | 5 | 5025487 | G | A_Leu | 564 | AA | rs41427849 | c.[2424+365G>A]+[2424+365G>A] | 3’UTR |
|  |  |  |  | B_Leu | 244 | AA | rs41427849 | c.[2424+365G>A]+[2424+365G>A] |  |
|  |  |  |  | A_Tu | 490 | AA | rs41427849 | c.[2424+365G>A]+[2424+365G>A] |  |
|  |  |  |  | B_Tu | 612 | AA | rs41427849 | c.[2424+365G>A]+[2424+365G>A] |  |
| **ING4** | 3 | 6632287 | T | A_Leu | 48 | CC | rs4764506 | c.[391+76T>C]+[391+76T>C] | Intron |
|  |  |  |  | A_Tu | 41 | CC | rs4764506 | c.[391+76T>C]+[391+76T>C] |  |
|  |  |  |  | B_Tu | 126 | CC | rs4764506 | c.[391+76T>C]+[391+76T>C] |  |
| **PTN** | 3 | 136563443 | A | A_Leu | 62 | GG | rs11505752 | c.[451-81A>G]+[451-81A>G] | Intron |
|  |  | 136590122 | G | A_Tu | 38 | TT | rs1162122 | c.[115+24G>T]+[115+24G>T] | Intron |
|  |  |  |  | B_Tu | 21 | TT | rs1162122 | c.[115+24G>T]+[115+24G>T] |  |
| **ZNF324** | 3 | 63674196 | A | A_Leu | 30 | GG | rs10418774 | c.[525A>G]+[525A>G] | synonymous |
|  |  |  |  | A_Tu | 45 | GG | rs10418774 | c.[525A>G]+[525A>G] |  |
|  |  |  |  | B_Tu | 67 | GG | rs10418774 | c.[525A>G]+[525A>G] |  |
| **RPL5** | 6 |  |  |  |  |  |  |  |  |
| **GPD1** | 5 |  |  |  |  |  |  |  |  |
| **CENPB** | 4 |  |  |  |  |  |  |  |  |
| **CIAPIN1** | 3 |  |  |  |  |  |  |  |  |
| **RPS2** | 3 |  |  |  |  |  |  |  |  |
| **RPS8** | 2 |  |  |  |  |  |  |  |  |
| **PDAP1** | 1 |  |  |  |  |  |  |  |  |
| **DDEF1** | 0 |  |  |  |  |  |  |  |  |

Blue = homozygous in all 4 samples; red = homozygous in 3 of 4 samples

*A_Tu = tumor DNA patient A, B_Tu = tumor DNA patient B; A_Leu = leukocyte DNA patient A, B_Leu = leukocyte DNA patient B ; **HGVC = Human Genome Variation Society
